# Supplementary material for: Extracted Plasma Cell-Free DNA Concentrations Are Elevated in Colic Patients with Systemic Inflammation
Source: Vet Sci. 2024 Sep 12;11(9):427. doi: 10.3390/vetsci11090427 (PMC11435807; doi:10.3390/vetsci11090427)
Supplement: Supplementary file 1 [file vetsci-11-00427-s001.zip › Table S1.pdf]

Bayless, RL; Cooper, BL; Sheats, MK.
Extracted plasma cell-free DNA concentrations are elevated in colic patients with systemic inflammation
Veterinary Sciences 2024

Supplemental Table S1. Median (range) cell-free DNA (cfDNA) concentrations in extracted plasma samples and extracted plasma cfDNA/blood segmented neutrophil ratios for groupings of colic patients. Significant differences between groups are indicated in the results section text and in figures.

	**Colic lesion category**			**Systemic Inflammatory Response Syndrome (SIRS) Status**		**Short-term Outcome**	
	Non-strangulating	Strangulating	Inflammatory	Non-SIRS	SIRS	Survival	Non-survival
**Plasma-extracted cell-free DNA concentration (ng/mL)**	2.9<br>(0.9-9.2)<br>[n = 38]	3.5<br>(0.9-41.6)<br>[n = 13]	2.2<br>(0.8-21.6)<br>[n = 12]	2.6<br>(0.8-6.8)<br>[n = 39]	3.0<br>(1.6-13.6)<br>[n = 21]	2.6<br>(0.8-13.6)<br>[n = 39]	3.3<br>(0.9-23.5)<br>[n = 25]
**Plasma-extracted cell-free DNA/ neutrophil ratio (ng/10^6 neutrophils)**	0.5<br>(0.2-1.2)<br>[n = 38]	0.5<br>(0.2-3.5)<br>[n = 11]	1.7<br>(0.1-6.77)<br>[n = 11]	0.5<br>(0.1-6.7)<br>[n = 39]	0.9<br>(0.2-5.3)<br>[n = 21]	0.5<br>(0.2-6.7)<br>[n = 40]	0.5<br>(0.1-5.3)<br>[n = 23]
