# Supplementary figures and images for: Extracted Plasma Cell-Free DNA Concentrations Are Elevated in Colic Patients with Systemic Inflammation
Source: Vet Sci. 2024 Sep 12;11(9):427. doi: 10.3390/vetsci11090427 (PMC11435807; doi:10.3390/vetsci11090427)

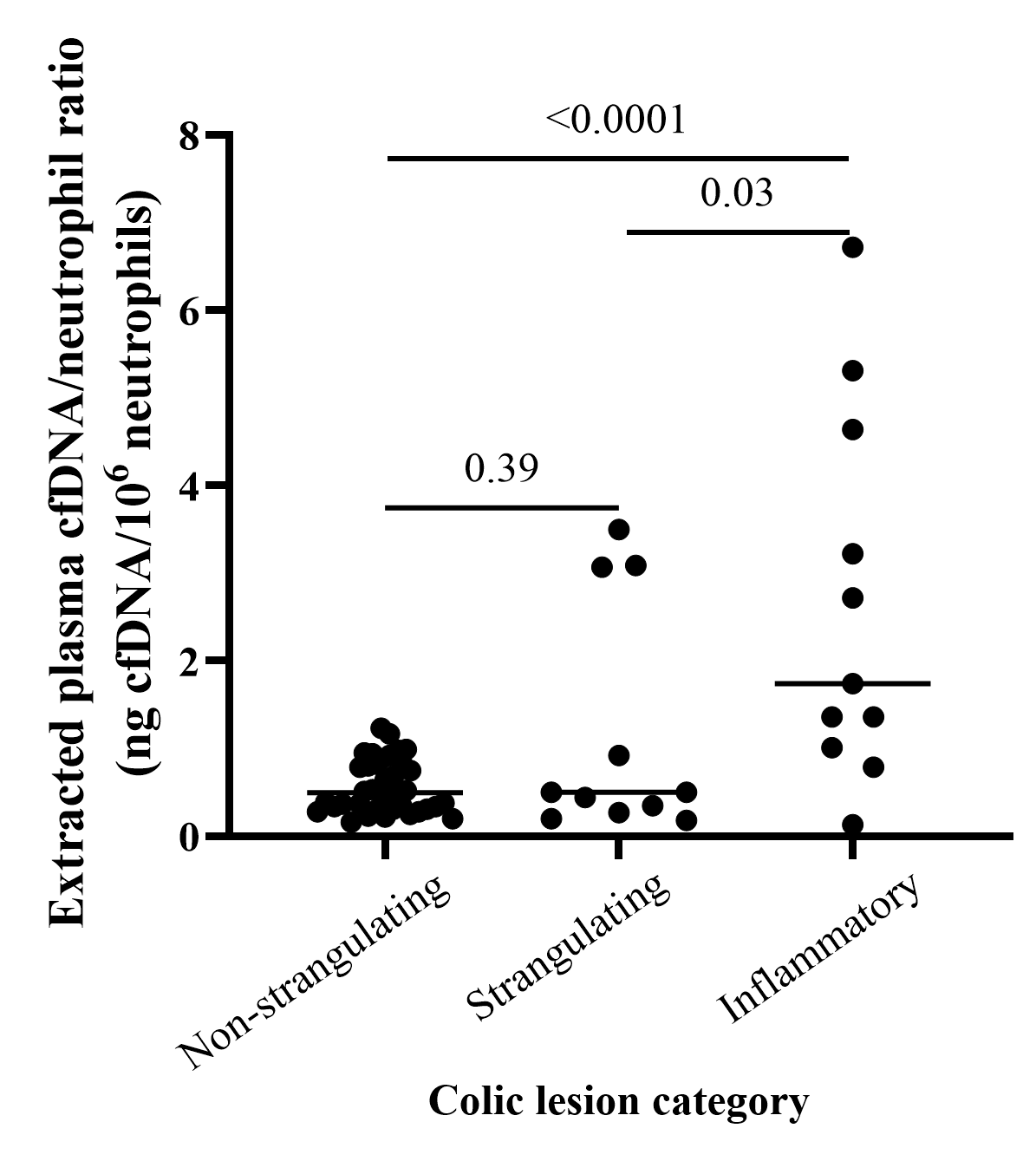

Supplement: Supplementary file 1 [file vetsci-11-00427-s001.zip › Figure S1.tif]

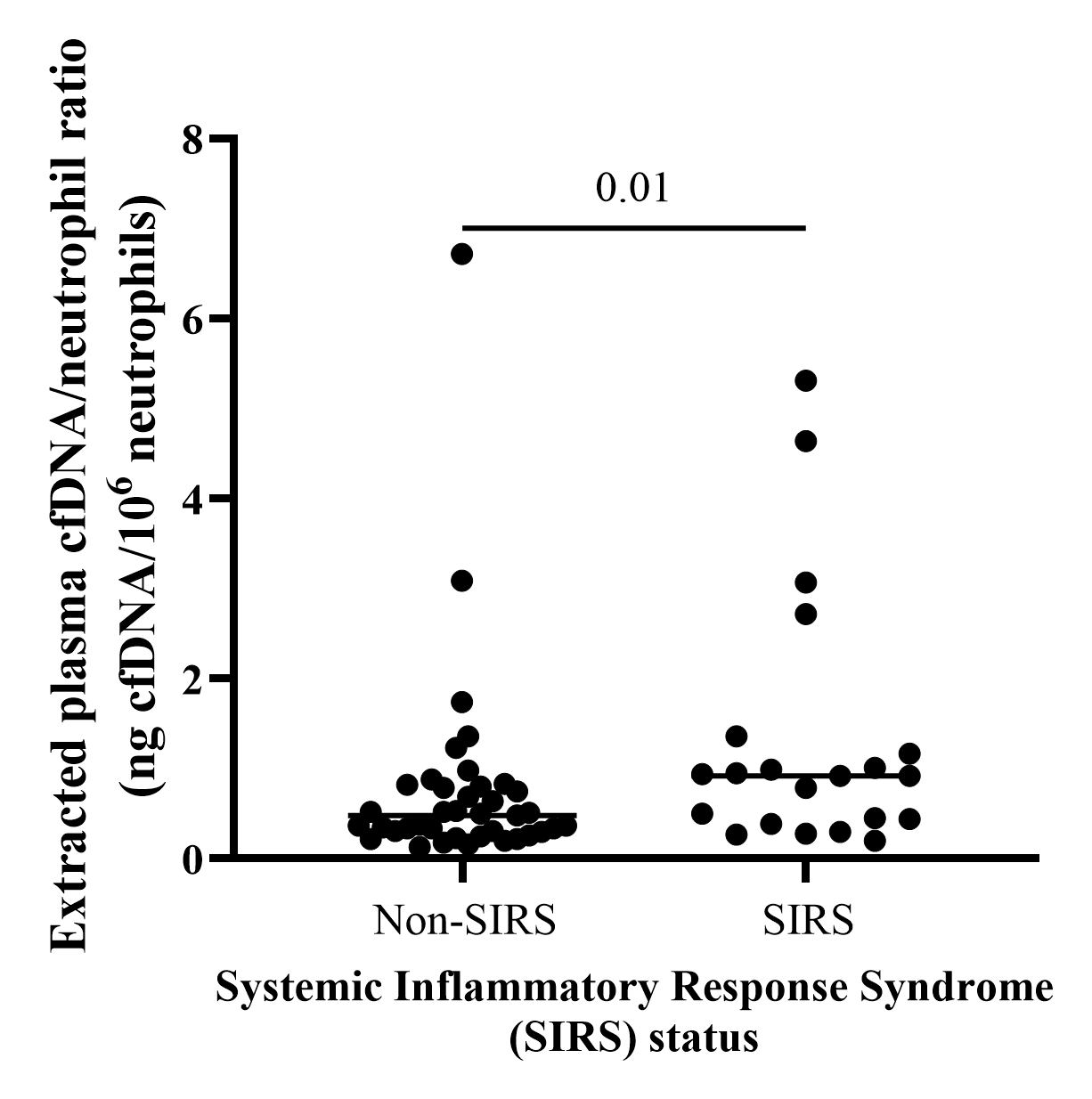

Supplement: Supplementary file 1 [file vetsci-11-00427-s001.zip › Figure S2.tif]

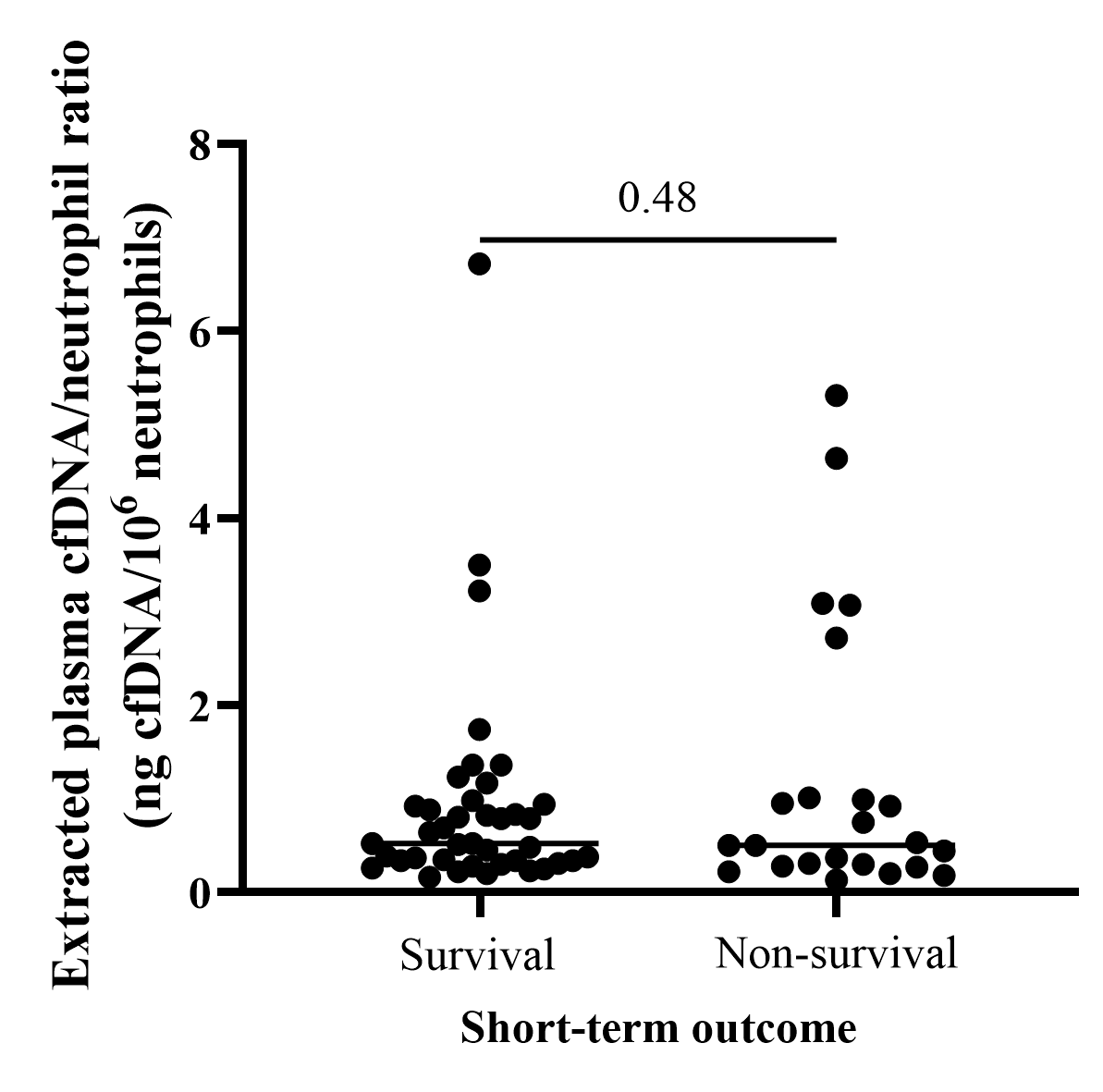

Supplement: Supplementary file 1 [file vetsci-11-00427-s001.zip › Figure S3.tif]
